# Long Island Veterinary Society

**Published:** 1890-06

**Authors:** D. S. Breslin

**Affiliations:** Secretary


*Long Island Veterinary Society.*—A regular meeting of the Long Island Veterinary Society was held on May 21st, 1890, at No. 74 Adam Street, the president, Dr. George H. Berns, in the chair. The following members were present: Drs. Berns, Atchison, Bell, Newman, Bowers, Buckley, Pendry, Breslin. The minutes of the previous meeting were read and approved. The Board of Censors made no report.

The Committee on Army Legislation reported progress. The Committee on State Legislation reported progress. A communication from Dr. John Lindsay, containing his resignation from the Society, was read, the reason assigned being his inability to attend the meetings of the Society. The Doctor residing in Huntington, L. I., found it impossible to attend meetings held in Brooklyn.

The next order of business being reading of papers, Dr. Samuel Atchison read an interesting paper entitled, "Professional Training and Ethics," which was generally discussed by the members, after which a vote of thanks was unanimonsly tendered to the essayist.

It was moved by Dr. Pendry, and seconded by Dr. Bowers, that the cost of printing reprints of the proceedings of the Society be held for future consideration. Carried. It was moved by Dr. Pendry, and seconded by Dr. Bell, that the Secretary communicate with the editor of the *American Veterinary Review* and editor of the JOURNAL OF COMPARATIVE MEDICINE AND VETERINARY ARCHIVES, notifying them that the paper read at this month's meeting of the Society would be withheld from publication, as its composition contained matters of a personal nature.

Bills for typewriting of last month's paper ($5.00), and fifty reprints of proceedings of Society ($6.00), amounting to eleven dollars, were ordered paid.

The chair appointed as essayist for June meeting Dr. Thomas M. Buckley. Meeting then adjourned. D. S. BRESLIN, D.V.S., *Secretary*.

